# Reshaping neuroimmunology: diagnosis and treatment in the era of precision medicine

**DOI:** 10.1055/s-0043-1777752

**Published:** 2023-12-29

**Authors:** Giordani Rodrigues dos Passos, Tarso Adoni, Maria Fernanda Mendes, Douglas Kazutoshi Sato

**Affiliations:** 1 Pontifícia Universidade Católica do Rio Grande do Sul, Escola de Medicina e Instituto do Cérebro do Rio Grande do Sul, Porto Alegre RS, Brazil.; 2 Universidade de São Paulo, Faculdade de Medicina, Hospital das Clínicas, São Paulo SP, Brazil.; 3 Santa Casa de São Paulo, Faculdade de Ciências Médicas, São Paulo SP, Brazil.

**Keywords:** Precision Medicine, Autoimmune Diseases of the Nervous System, Multiple Sclerosis, Neuromyelitis Optica, Biomarkers, Immunomodulation, Pharmacogenetics, Medicina de Precisão, Doenças Autoimunes do Sistema Nervoso, Esclerose Múltipla, Neuromielite Óptica, Biomarcadores, Imunomodulação, Farmacogenética

## Abstract

Precision medicine has revolutionized the field of neuroimmunology, with innovative approaches that characterize diseases based on their biology, deeper understanding of the factors leading to heterogeneity within the same disease, development of targeted therapies, and strategies to tailor therapies to each patient. This review explores the impact of precision medicine on various neuroimmunological conditions, including multiple sclerosis (MS), neuromyelitis optica spectrum disorder (NMOSD), myelin oligodendrocyte glycoprotein antibody-associated disease (MOGAD), optic neuritis, autoimmune encephalitis, and immune-mediated neuropathies. We discuss advances in disease subtyping, recognition of novel entities, promising biomarkers, and the development of more selective monoclonal antibodies and cutting-edge synthetic cell-based immunotherapies in neuroimmunological disorders. In addition, we analyze the challenges related to affordability and equity in the implementation of these emerging technologies, especially in situations with limited resources.

## Resumo

A medicina de precisão está revolucionando o campo da neuroimunologia, com uma abordagem inovadora caracterizada pela classificação de doenças com base em sua biologia, compreensão mais profunda dos fatores que levam à heterogeneidade dentro da mesma doença, desenvolvimento de terapias com alvos específicos e estratégias para adaptar as terapias a cada paciente. Esta revisão explora o impacto da medicina de precisão em várias condições neuroimunológicas, incluindo esclerose múltipla (EM), distúrbio do espectro da neuromielite óptica (NMOSD), doença associada ao anticorpo anti-glicoproteína da mielina do oligodendrócito (MOGAD), neurites ópticas, encefalites autoimunes e neuropatias imunomediadas. Discutimos avanços na subclassificação de doenças, reconhecimento de novas entidades, biomarcadores promissores e desenvolvimento de anticorpos monoclonais mais seletivos e imunoterapias de ponta baseadas em células sintéticas para as condições acima. Além disso, analisamos os desafios relacionados com acessibilidade e equidade na implementação dessas tecnologias emergentes, especialmente em ambientes com recursos limitados.

## INTRODUCTION

**Figure 1:**
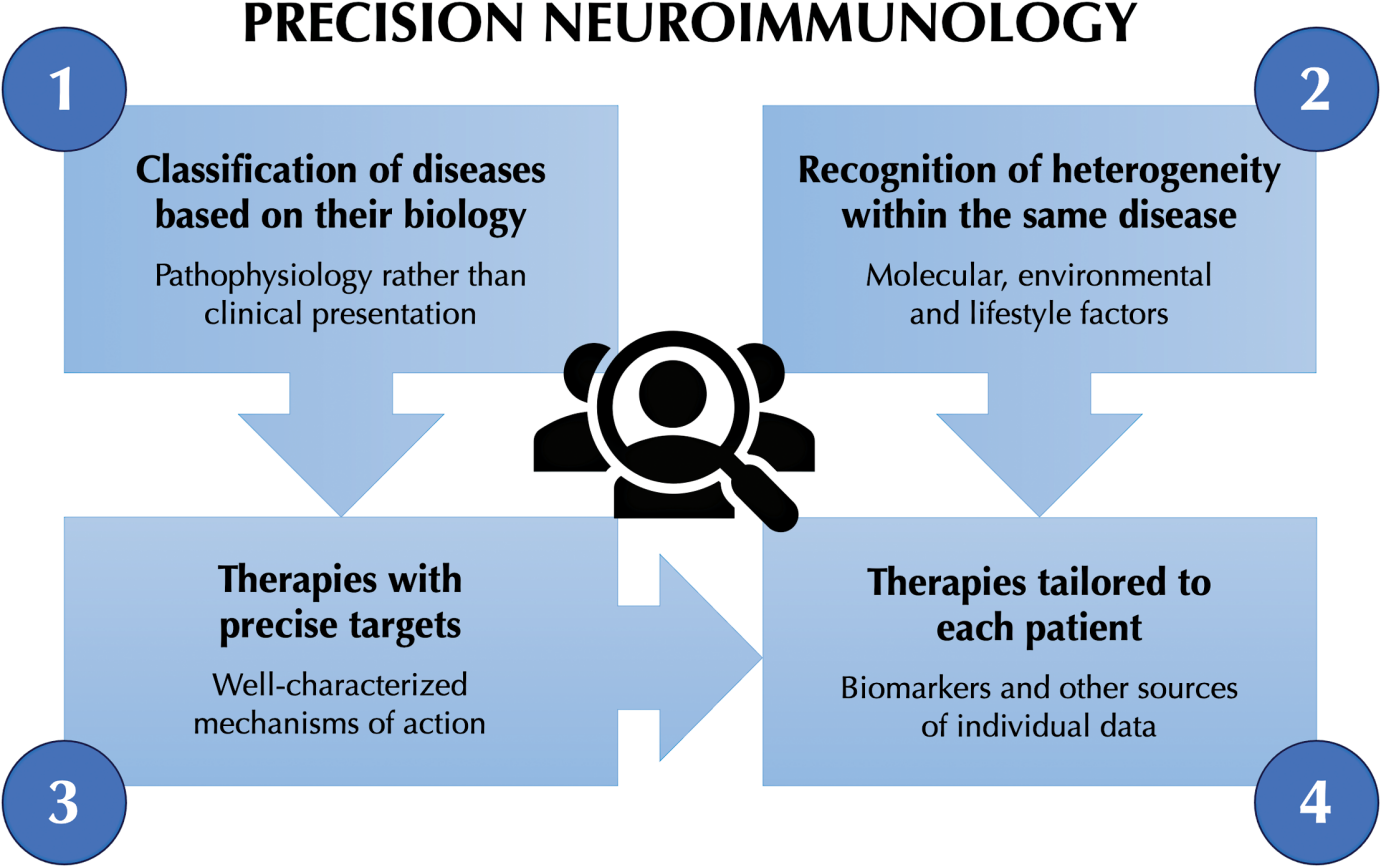
Schematic representation of the main concepts encompassed by precision medicine and applicable to neuroimmunology.

With the ever-growing arsenal of biomarkers and targeted therapies available to assess and treat neuroinflammatory conditions, precision medicine has paved its way into the field of neuroimmunology. This approach encompasses:

- classifying diseases based on their biology, rather than on clinical presentation alone,
- recognizing the molecular, environmental, and lifestyle factors that account for heterogeneity within the same disease,
- moving towards therapies with precise targets and well-characterized mechanisms of action, and
- tailoring therapies to each patient based on biomarkers and other sources of individual health data 1 ( Figure 1 ).

This approach emerged first in oncology and genetics and is now popular across a range of fields in medicine.

In this review, we discuss how this evolving paradigm is already changing the way we approach conditions like multiple sclerosis (MS), neuromyelitis optica spectrum disorder (NMOSD), myelin oligodendrocyte glycoprotein antibody-associated disease (MOGAD), optic neuritis (ON), autoimmune encephalitis, and immune-mediated neuropathies in the clinical practice. We discuss the recognition of novel entities and the reclassification of existing conditions, the development of novel biomarkers and targeted drugs, and some of the challenges of incorporating these novel technologies into clinical practice in the field of Neuroimmunology. In Box 1 , we compare the conventional approach with the emerging, precision-based approach to the management of neuroimmunological conditions based on illustrative cases.

**Box 1:** Comparison of the conventional approach with the emerging, precision-based approach to the management of neuroimmunological conditions based on three illustrative cases.

Conventional approach	Precision medicine approach
An 18-year-old female presents with transverse myelitis extending from T6 to the conus medullaris and recovers well following intravenous steroids. Retrospectively, she reports an episode suggestive of unilateral optic neuritis at age 16, with spontaneous recovery of visual acuity but with residual dyschromatopsia. Testing for AQP4-IgG results negative and a hypothesis of seronegative NMOSD is made, but she fails to meet the diagnostic criteria. Prednisone and azathioprine are started off-label.	Following the two attacks, the same patient is tested for AQP4-IgG and MOG-IgG using live cell-based assays, with the later coming out positive at high titer. A diagnosis of MOG-IgG-associated disease (MOGAD) is made, and the patient is enrolled into one of the ongoing, phase 3 clinical trials testing selective monoclonal antibodies against either the interleukin-6 receptor (satralizumab) or the neonatal Fc receptor (rozanolixizumab). Serial MOG-IgG testing is performed during follow-up.
A 53-year-old male is admitted to the intensive care unit with a four-week history of symmetric ascending weakness and pain, tremor, ataxia, bilateral facial palsy, and eventually respiratory insufficiency. He receives intravenous immunoglobulin due to suspected GBS. Since his condition continues to deteriorate another four weeks later, CIDP is suspected, and indeed he fulfills the EFNS/PNS electrophysiological diagnostic criteria. However, he fails to respond to intravenous methylprednisolone and then to plasma exchange and is discharged home with tracheostomy and unable to walk.	Soon after admission, the presence of tremor and ataxia prompts testing for IgG antibodies against CASPR1/contactin-1 complex, which come out positive, allowing for a diagnosis of paranodopathy instead of CIDP. Following failure of intravenous immunoglobulin, he receives rituximab (instead of intravenous steroids or plasma exchange) and presents good recovery of respiratory and motor function (requires some help for usual activities, but is able to walk unassisted at discharge).
A 38-year-old female, who had been on teriflunomide since age 32 after receiving a diagnosis of relapsing-remitting MS, presented with a two-week complaint of paroxysmal paresthesia on the left side of her body, including the face, superimposed to increasing fatigue over the past six months. Neurological examination remains unchanged and six-monthly 1.5 Tesla MRI scans of the brain have not shown any new lesions since the start of teriflunomide. Her neurologist suggests the paresthesia may represent a pseudorelapse or a non-organic symptom, given the absence of MRI activity, and explains to her there are no grounds to consider a DMT switch.	To further assess the new symptoms, the treating neurologist orders a 3.0 Tesla MRI of the brain, which shows two paramagnetic rim lesions, and measurements of serum neurofilament light chain, which comes out elevated (Z score = 1.65). She explains to the patient these biomarkers are not yet fully validated, but likely suggest the presence of chronic active disease and increased risk for future disease activity. Taking these findings into account, the neurologist ends up considering the paresthesia as a probable true relapse, in spite of no new MRI lesion, and raises the possibility the worsening fatigue may represent smoldering MS. A shared decision is made to switch to a higher efficacy DMT.

Abbreviations: CIDP, chronic inflammatory demyelinating polyradiculopathy; DMT, disease-modifying therapy; EFNS/PNS, European Federation of Neurological Societies/Peripheral Nerve Society; GBS, Guillain-Barré syndrome; MOGAD, myelin oligodendrocyte glycoprotein antibody-associated disease; MRI, magnetic resonance imaging; MS, multiple sclerosis; NMOSD, neuromyelitis optica spectrum disorder.

## REDEFINING DISEASE CLASSIFICATION

### MS phenotypes

Precision medicine has influenced not only how we define nosological entities, but also the classification of disease subtypes, with MS as an example. Historically, the classification of MS into phenotypes has been done purely on clinical grounds: relapsing disease (clinically isolated syndrome [CIS] or relapsing-remitting MS [RRMS], resulting from focal inflammatory activity) or progressive disease (either primary [PPMS] or secondary [SPMS], resulting mainly from neurodegeneration). 2 However, biological processes typically associated with progression (such as accelerated brain atrophy) may appear since the early stages of relapsing disease, even before the first relapse (e.g., in patients with radiologically isolated syndrome). On the other hand, focal inflammatory activity, either silent or in the form of relapses, may occur during progressive disease. Clinically, MS is very heterogeneous and originates from different biological disease processes. Therefore, a biological classification of MS is needed to integrate different types of therapeutic interventions.

A more modern understanding is the view of MS not as four distinct phenotypes, but rather as a continuum of different biological processes occurring concurrently, at varying degrees over time and across individuals. 3 This concept is in line with precision medicine approaches and may influence future revisions of MS classification, therefore affecting the selection of patients for clinical trials, regulatory approval of therapies, and treatment algorithms.

Under precision medicine approaches, emphasis is put on incorporating biomarkers to accurately identify the biological processes taking place in a given time at a given patient (focal inflammatory activity, widespread low-grade inflammation, neuroaxonal loss, remyelination failure, etc), as well as quantifying each of them in order to understand their relative contribution to disease pathogenesis and track them over time. 4 An early step towards this was the introduction of the so-called phenotype modifiers (active versus not active, and with versus without progression) in the 2013 revision, acknowledging the coexistence of relapsing activity and progression and incorporating the use of biomarkers to define disease activity – gadolinium-enhancing lesions or new or unequivocally enlarging T2 lesions on magnetic resonance imaging (MRI). 2

It is not unrealistic to suppose that, in the future, further phenotype modifiers based on biomarkers for chronic perilesional inflammation, neuroaxonal degeneration, and remyelination (see section “Novel Biomarkers”) could be incorporated to allow improved subtyping of MS. 4 For instance, serum neurofilament light chain (sNfl) could also contribute to the classification of MS as active or not active, whereas measurement of brain atrophy on MRI and retinal nerve fiber layer thickness on optical coherence tomography could contribute to identify ongoing progression. 2 4 Such refinements in MS classification will hopefully allow for improvements in personalized therapy.

### MOGAD

Following the description of the then-called NMO-IgG in 2004 and the identification of aquaporin-4 as its target in 2005, aquaporin-4 antibody (AQP4-IgG) emerged as a biomarker for neuromyelitis optica, being found in most but not all patients with this condition. 5 Later, an antibody against myelin oligodendrocyte glycoprotein (MOG-IgG) was found in a subset of patients previously diagnosed with neuromyelitis optica who were seronegative for AQP4-IgG and presented less female predominance, more frequent involvement of optic nerve and conus medullaris, higher proportion of monophasic disease and better recovery following attacks. 6 7 MOG-IgG was also linked to phenotypes not usually seen in NMOSD, such as acute disseminated encephalomyelitis (ADEM) and cerebral cortical encephalitis. 8

Patients with MOG-IgG differ from those with AQP4-IgG not only in terms of demographics, clinical presentation, and disease course, but also regarding pathophysiology and response to treatment: whereas MOG-IgG correlates with myelin damage, AQP4-IgG leads to an astrocytopathy with complement activation and secondary demyelination. 9 Patients with MOG-IgG are frequently responsive to corticosteroids in the acute phase and less responsive to rituximab as maintenance therapy, whereas those with AQP4-IgG usually require second-line acute treatments such as plasma exchange and respond quite well to rituximab in the long term. 10

All these differences led to the recognition of MOGAD as a separate entity, culminating in the International MOGAD Panel proposed criteria in 2023. 8 Of note, unlike in MS or NMOSD, the positivity of a biomarker (MOG-IgG) is indispensable for the fulfillment of the diagnostic criteria, making this a good example of the precision medicine approach. Likewise, MOG-IgG positivity is also crucial for inclusion into the first phase 3 clinical trials in MOGAD, which are ongoing to investigate the safety and efficacy of rozanolixizumab (ClinicalTrials.gov ID NCT05063162) and satralizumab (NCT05271409) and will likely be a requirement for accessing these therapies in the future in case they get marketing approval. In line with this, the importance of implementing validated, gold-standard assays for antibody detection in clinical practice cannot be understated.

On the other hand, an overreliance on biomarkers can be hazardous for other reasons. The indiscriminate order of serum or CSF autoantibodies is associated not only with increasing costs but also with false positives and their consequences. Jarius et al illustrate this by calculating that, assuming a hypothetical prevalence of 1% of MOGAD among cases currently diagnosed with MS, screening all MS cases with a hypothetical MOG-IgG assay with 100% sensitivity and 99% specificity would produce 1000 true positive results alongside 990 false positive results, which would be unacceptable. 11 Therefore, MOG-IgG testing should be reserved for patients with a compatible clinical and radiological picture and low positive results should be interpreted with caution. 8

### Optic neuritis

Diagnostic criteria and a novel classification system for ON subgroups have been proposed by an international panel in 2022, based not only on paraclinical tests, such as autoantibodies, MRI, and OCT, but also on a precise characterization of clinical presentation, course, and medical history. 12 This highlights the continuing importance of semiology skills in the era of precision medicine to reduce the risk of misdiagnosis and aid in therapeutic decisions.

With the new system, loss of vision can be diagnosed as definite ON, possible ON, or not ON. 12 Then, ON can be dichotomized into autoimmune (usually relapsing) or infectious or systemic (usually monophasic) – this is the level 1 classification, to guide general management. In level 2 classification, autoimmune cases can be further divided according to the specific etiology or a phenotypic category. Finally, level 3 classification deals with anecdotal causes of ON for which no consensus has been reached yet. Furthermore, this novel system introduces an anatomical classification including whole-body, brain, orbital, and prelaminar compartments.

Three validated biomarkers have been included to define specific categories in level 2 classification: AQP4-IgG, MOG-IgG, and the paraneoplastic CRMP5-IgG (also known as CV2-IgG). 12 Obviously, MS-ON also represents a specific category. In addition, the panel highlights that ON is associated with more disorders than previously thought, with the remaining autoimmune cases subdivided for now into single isolated ON, relapsing isolated ON, chronic relapsing inflammatory optic neuropathy, prelaminar ON, and primary progressive ON. 12

### Autoimmune encephalitis

Few conditions in neuroimmunology have undergone such an extensive nosological reclassification in recent years as autoimmune encephalitis. That has been driven by the description of many novel autoantibodies, each defining a specific type of encephalitis, even when there might be some overlap in terms of clinical phenotypes.

In 2010, it became clear that, amongst antibodies targeting the voltage-gated potassium channel (VGKC) complex, only those whose antigenic targets were in the extracellular domains were consistently associated with neuroimmunological conditions. 13 These antibodies against the VGKC complex were later identified as antibodies against leucine-rich glioma-inactivated 1 (LGI1-IgG) and contactin-associated protein-like 2 (CASPR2-IgG). Routine use of assays detecting these antibodies allowed the identification of their respective manifestations: some common to both, such as focal seizures, amnesia, dysautonomia, neuromyotonia, and neuropathic pain, and some exclusive of LGI-IgG, such as faciobrachial dystonic seizures. 13 This illustrates how the same autoantibody can produce distinct phenotypes.

On the other hand, distinct autoantibodies can lead to a common phenotype, as in the case of limbic encephalitis. A modern view is that rather than a single disease, limbic encephalitis is a manifestation that can develop as a result of (or at least in association with) several autoantibodies against neuronal surface antigens (such as LGI1, CASPR2, AMPA, and GABA _B_ ) or intracellular antigens (such as Hu [ANNA1], Ma2, and GAD). 14 Identifying the autoantibody involved in a given case has practical implications, as it helps define the risk (and most likely site) of an associated neoplasm as well as response to immunotherapy and prognosis.

Although autoantibody testing is crucial for the precision management of autoimmune encephalitis, a significant proportion of cases remain negative for all antibodies included in commercially available panels. These cases may be challenging to classify, but clues from diligent clinical observation as well as clinical criteria are available to guide proper diagnosis. 15 Attention is needed to avoid misdiagnosis of autoimmune encephalitis based on positive results from autoantibody panels requested in the absence of clear indication to test, or with poorly significant results (such as low-titer anti-GAD) or using older assays with limited validity (such as anti-VGKC). Indeed, proper interpretation of the available biomarkers is of paramount importance in the era of precision medicine.

### Immune-mediated neuropathies

Historically, immune-mediated neuropathies have been classified on clinical and electrophysiological grounds (e.g., sensory and/or motor, time to nadir, demyelinating versus axonal) and named with eponymous or purely descriptive umbrella terms, such as Guillain-Barré syndrome (GBS) and chronic inflammatory demyelinating polyneuropathy (CIDP). The fact that several variants have been described and that treatment response is variable suggests significant heterogeneity in terms of pathophysiology, even amongst patients grouped under the same diagnostic label.

Over the past decade, the identification of autoantibodies targeting nodal/paranodal antigens has allowed for the recognition of distinct entities and a more precise classification. Interestingly, the group of so-called nodo-paranodopathies include conditions that may present with demyelinating features on nerve conduction studies despite having no evidence of demyelination on pathology; also, others may have electrophysiological features suggestive of axonal neuropathy yet present prompt recovery after treatment, which would be unlikely in case of true axonal pathology. 16 These apparent inconsistencies highlight the need for a more biological classification of these conditions.

A sectorial/antigenic classification of immune-mediated neuropathies has been proposed based on the involved domain(s) of the myelinated fiber, and, when known, the target antigen. 17 For instance, antibodies against neurofascin-140/186 (in the node) or neurofascin-155 or contactin-1 (both in the paranode) could produce a clinical picture resembling CIDP, whereas antibodies against GM1 (in the node-paranode) could be associated with either acute motor and sensory axonal neuropathy or multifocal motor neuropathy. Several other autoantibodies against nodal/paranodal antigens are already known and more are likely to be discovered in the future.

The modern classification of immune-mediated neuropathies is not a matter of nosological taxonomy only. It promotes precision by identifying subgroups with distinct phenotypes and specific management needs. For instance, those with nodal/paranodal antibodies tend to have a more rapidly progressive, severe, motor- and distal-predominant polyneuropathy, initially resembling GBS but later meeting the criteria for CIDP, but often with associated tremor or ataxia, who respond poorly or only transiently to the usual immunomodulatory therapies (intravenous immunoglobulin, plasma exchange, and corticosteroids) and are likely to benefit from less conventional therapies (such as rituximab). 16

## NOVEL BIOMARKERS

### MRI and PET imaging in MS

MRI continues to evolve as the most important tool in the diagnosis and monitoring of MS. Novel markers have emerged, such as paramagnetic rim lesions (PRLs) and slowly expanding lesions (SELs). 18 The former are detected by susceptibility-weighted MRI, whereas the latter are detected by longitudinal assessment of white matter lesions on volumetric MRI using automated techniques. Frequently found in progressive MS, both PRLs and SELs denote chronic active lesions, that persist well beyond the acute phase and cause ongoing tissue injury. These markers illustrate how MRI will allow for progressively better *in vivo* inferences about pathological aspects of disease.

Positron emission tomography (PET), in turn, enables the assessment of more specific processes of MS, owing to the use of radiolabeled compounds that bind to specific targets. For instance, 18-kDa translocator protein (TSPO) tracers identify neuroinflammation in the form of activated macrophages and microglia; ^11^ C-PiB, ^18^ F-florbetaben, and ^18^ F-florbetapir enable measurement of demyelination and remyelination; and ^18^ F- FDG provides an indirect measure of neuronal damage. 19 Systems wherein MRI replaces computed tomography decrease the total dose of ionizing radiation involved in PET studies. 19 Therefore, PET is likely to become more and more popular as a tool to aid the development and implementation of precision medicine strategies.

### Fluid biomarkers to monitor disease activity

Not only diagnosis but also prognostication and treatment response can be aided by biomarkers. This is a mainstay in precision medicine, if one aims to optimize the selection of patients who need long-term immunosuppression or could benefit from a particular mechanism of treatment, or for identifying treatment failure prior to the occurrence of a relapse.

In MS, serum neurofilament light chain (sNfl) is emerging as the most promising biomarker apart from MRI. Although not specific to MS, under this condition, it reflects ongoing neuroaxonal damage thought to result mainly from relapses and/or new MRI lesions. 20 It has been suggested sNfl could aid in estimating the risk of conversion to MS in patients with radiologically isolated syndrome, establishing the diagnosis of MS, distinguishing its subtypes, predicting further relapses and new MRI lesions in patients with relapsing MS, and monitoring disease activity during treatment with DMTs (which could be particularly useful for patients with contraindications to MRI). 20 21 As commercial assays are increasingly available, sNfl is gradually being incorporated into clinical practice, although algorithms on how it can be used to influence treatment decisions on an individual level still need further validation. Its incorporation into clinical trials as a secondary endpoint (which occurred first in the phase 3 trial of ofatumumab versus teriflunomide in relapsing MS) will help establish its validity as a biomarker in MS. 22

Serial autoantibody testing may have a role in monitoring antibody-mediated diseases. In MOGAD, reduction of MOG-IgG titers or conversion to seronegative status is associated with a lower risk of further attacks, particularly in children and within 2 years from the first attack. 23 24 25 In AQP4-IgG NMOSD, the association between antibody titers and disease activity is less clear, with some studies showing no prognostic or predictive utility 26 and others suggesting that unchanged or increased AQP4-IgG titers during treatment with immunosuppressants is a risk factor for relapse. 27

Serum glial fibrillary acidic protein (GFAP) – a marker of astroglial damage – is emerging as the most relevant biomarker for longitudinal monitoring of NMOSD, as it predicts attack risk, increases during attacks and correlates with attack severity. 28 29 Interestingly, in the inebilizumab phase 3 trial, serum GFAP was reduced during treatment even in patients who did not experience attacks during the study, serving as a marker of treatment effect. 28

In addition to body fluid and imaging biomarkers, promising novel ways to assess MS include the so-called digital biomarkers, such as wearable sensors and smartphone applications, as well as patient-reported outcomes. 30 These tools can generate large volumes of data on aspects of disease that are not fully captured by conventional tools and may further inform precision medicine approaches.

### Pharmacogenomics to predict safety and efficacy

Although several disease-modifying therapies (DMTs) are approved for MS, our ability to predict their efficacy, safety, and tolerability at the individual level is still limited. Attempts to incorporate pharmacogenomics to overcome this issue have been made since the time when interferon-beta and glatiramer acetate were the only DMTs available. 31 However, it was only recently, with the approval of siponimod, that pharmacogenomic testing made its way into MS clinical practice.

Siponimod is a sphingosine 1-phosphate (S1P) receptor modulator, with a similar mechanism of action to fingolimod but more selective, resulting in an improved safety profile. Single nucleotide polymorphisms in the CYP2C9 gene affect an individual's ability to metabolize this drug, influencing its plasmatic levels and therefore safety, but not effectiveness. 32 The approved label mandates CYP2C9 genotyping prior to initiation of siponimod. For patients carrying a CYP2C9*3/*3 genotype (around 0.3% to 0.4% of Caucasians and less in other ethnic groups) the drug is formally contraindicated, whereas for those with the CYP2C9*1/*3 and CYP2C9*2/*3 genotypes the daily maintenance dose must be 1 mg instead of 2 mg. 32 Other polymorphisms in the CYP2C9 are under investigation regarding their influence on the safety of siponimod. 33

Several single nucleotide polymorphisms are possible candidates to predict the effectiveness of other DMTs. Amongst those investigated in MS, the most promising are in the GSTP1, ITGA4, NQO1, AKT1, and GP6 genes (for natalizumab), ZMIZ1 (for fingolimod and dimethyl fumarate), NOX3 (for dimethyl fumarate), and ADA (for cladribine). 32 However, further validation is needed before they can be incorporated into clinical practice.

In NMOSD, the effectiveness of rituximab – an anti-CD20 monoclonal antibody – is affected by polymorphisms in the FCGR3A gene that influence the binding affinity between this drug and the low-affinity Fc-γ receptor IIIa in their target cells. 34 This explains why some patients fail to achieve sufficient CD20+ B cell depletion and remain therefore prone to attacks despite treatment with rituximab. Based on this, one of the first DMTs developed specifically for NMOSD – inebilizumab, an anti-CD19 monoclonal antibody – has been engineered on purpose to allow for enhanced binding to the Fc-γ receptor IIIa, to make its effectiveness less dependent on the FCGR3A genotype. 35 This illustrates how precision medicine can inform the development of drugs with higher response rates and suitable for a wider range of patients.

The efficacy of eculizumab and ravulizumab, two anti-C5 monoclonal antibodies approved for NMOSD and MG, may also be influenced by genetics. A minority of patients, usually of Japanese descent, can harbor a polymorphism in the gene encoding C5, which makes it resistant to blockade by eculizumab and, theoretically, by ravulizumab as well. 36 Under a precision medicine approach, physicians will be able to identify those rare patients who would fail to respond to such a highly effective (and costly) drug, preventing unwanted attacks and costs.

## NOVEL TREATMENTS

### On-label drugs for NMOSD

Apart from rituximab, all drugs available to treat NMOSD prior to 2019 were wide-spectrum immunosuppressants, like oral steroids, azathioprine, and mycophenolate. They had several side effects, limited efficacy, and/or long latency to onset of clinical effect. Fortunately, growing knowledge of the pathophysiology of NMOSD led to the development of the first on-label, targeted therapies, with four drugs approved for NMOSD between 2019 and 2023.

Inebilizumab targets the same subset of B cells and B cell precursors targeted by rituximab plus CD19+ plasmablasts and plasma cells, that produce AQP4-IgG. 37 Satralizumab (an anti-interleukin-6 [IL6] monoclonal antibody) inhibits the differentiation of B cells into plasma cells and consequently the production of IgG (including AQP4-IgG). 38 39 Eculizumab and ravulizumab bind to the terminal complement component C5 preventing its cleavage into C5a and C5b, thus blocking complement-dependent cytotoxicity, which is one of the main effects produced by AQP4-IgG after binding to astrocytes. 40 41

In common, all the novel drugs have precise targets, well-characterized mechanisms of action, very high efficacy in increasing the time to a first relapse (primary endpoint in all the trials), and fewer side effects than wide-spectrum immunosuppressants. 42 However, since all approvals have been for AQP4-IgG-seropositive NMOSD, the treatment of seronegative patients remains a challenge.

### Bruton tyrosine kinase (BTK) inhibitors for MS

In MS, the lack of a known putative autoantigen and the heterogeneity of pathogenic mechanisms across patients and disease stages make it more challenging to develop targeted therapies such as those developed for NMOSD. Even the disease-modifying therapies (DMTs) that are most effective in terms of controlling relapsing activity – monoclonal antibodies such as natalizumab, alemtuzumab, ocrelizumab, and ofatumumab – are ineffective or only modestly effective in controlling MS progression, presumably due to their lack of effect on CNS-compartmentalized B cells and microglia. 43

In this context, the class of BTK inhibitors has emerged as a promising option. These are CNS-penetrant molecules that can exert effects on both adaptive and innate immune cells, outside and within the CNS. According to preclinical studies, they modulate the activation of B cells and microglia, infiltration of lymphocytes into the CNS, leptomeningeal inflammation, demyelination, and remyelination. 43 44

Clinical trials in relapsing and/or progressive forms of MS are ongoing with six BTK inhibitors: evobrutinib, tolebrutinib, fenebrutinib, remibrutinib, orelabrutinib and BIIB091. 45 Results of phase 2 trials have been published for the former two and showed a reduction of relapsing activity and generally favorable safety profile. 46 47 The ongoing phase 3 trials shall inform whether this class is really safe and effective, particularly for progressive disease. Anyway, BTK inhibitors do represent a new mechanism to treat MS, beyond those offered by current DMTs. If phase 3 trials result positive, these drugs will improve our ability to address the distinct mechanisms of MS and personalize therapy, in line with the promise of precision medicine.

### Synthetic cell-based immunotherapies

Chimeric antigen receptor (CAR) T-cells are synthetic, molecularly precise immunotherapies, that showed remarkable success in the treatment of some hematological malignancies. They are produced from T-cells collected from the patient by leukapheresis and genetically reengineered in the laboratory to express CARs that recognize specific target antigens; these cells are then proliferated ex-vivo and manufactured as an autologous product that is reinfused into the patient. 48 This innovative technology is now under investigation in neuroimmunological disorders, with targets defined based on precise knowledge of disease pathophysiology.

In a small phase 1b/2a trial, CAR T-cells against the B-cell maturation antigen (BCMA), which specifically targets plasma cells, seemed safe and well tolerated and led to clinically meaningful improvement of disease severity in patients with generalized myasthenia gravis (MG); surprisingly, there was no evidence of hypogammaglobulinemia or increased susceptibility to infection. 49 A small phase 1 trial in AQP4-IgG-seropositive NMOSD also showed a manageable safety profile and therapeutic potential for anti-BCMA CAR T-cells. 50 Unlike monoclonal antibodies, CAR T cells seem to be able to cross the blood-brain barrier. 48 Indeed, in a B-cell–dependent experimental autoimmune encephalomyelitis (EAE) model, anti-CD19 CAR T-cell therapy led to B-cell depletion not only in peripheral tissues but also in the CNS and amelioration of EAE. 51

A modification from the original CAR T-cell therapy strategy is chimeric *autoantibody* receptor (CAAR) T-cell therapy, which targets autoantigen-specific B cells. 48 A phase 1 trial of CAAR T cells against memory B cells that produce MuSK-IgG is ongoing in MuSK-IgG-seropositive MG (NCT05451212), whereas CAAR T cells targeting the NMDA receptor (potentially useful for NMDAr-IgG autoimmune encephalitis) have been developed but not yet moved into clinical testing. 48 For the foreseeable future, such a level of selectivity is arguably the best one can imagine in the treatment of autoimmune diseases and illustrates the potential of precision medicine.

## CHALLENGES FOR IMPLEMENTATION

With many unmet needs remaining in neuroimmunological disorders, neurologists and patients are eager to access the diagnostic tools and therapeutic strategies that characterize precision medicine. Although many of these resources hold the potential to improve outcomes, not all of them are cost-effective at present; in fact, some are posing huge pressure on healthcare systems. In this context, precision medicine efforts must necessarily consider aspects like affordability and equity.

The development of innovative drugs for rare diseases is needed but comes at a huge cost and is already leading to a sustainability crisis. Solutions might include adaptive clinical trial designs to reduce the time and costs of drug development, government-sponsored clinical trials to seek regulatory approval for drugs that may no longer be commercially appealing for pharmaceutical companies and incorporation of novel treatments under risk-sharing agreements. 52

Furthermore, since the provision of personalized care is a core purpose of precision medicine, an equity agenda is needed to ensure minority populations are also represented in clinical trials and studies involving genetics, for instance. 53 54 Likewise, efforts should be made to ensure reasonable access to relevant biomarkers and approved therapies across the different geographical regions and healthcare systems, rather than in a few research centers in resource-rich areas.

Lastly, but equally important, is the urgent need for practitioners to preserve and refine their clinical observation and diagnostic reasoning skills, which cannot be replaced by any of the sophisticated tests currently available. Even in the era of precision medicine, novel technologies should only be incorporated as part of rational algorithms, following proper consideration of clinical aspects and theoretical evidence.

In conclusion, precision neuroimmunology is no longer a mere future perspective. Identification of novel disease entities such as MOGAD and several autoimmune encephalitides and reclassification of existing ones such as immune-mediated neuropathies have significantly changed the taxonomy of neuroimmunological diseases. Novel biomarkers are being incorporated into clinical practice and novel drugs have been developed with precise targets for diseases like MS and NMOSD, using either established technologies (such as monoclonal antibodies) or highly innovative platforms (such as CAR or CAAR T-cells). Countless challenges remain, especially related to affordability and equity of the newer technologies. In the future, we should ensure that patients can benefit from the unprecedented possibilities of this novel approach.
